# Internationalization of Multinational Companies and Cognitive Differences Across Cultures: A Neuroeconomic Perspective

**DOI:** 10.3389/fpsyg.2022.807582

**Published:** 2022-03-03

**Authors:** Huifang Cheng, George Kwame Agbanyo, Tianlun Zhu, HuiHong Pan

**Affiliations:** ^1^School of Economics, Zhejiang University of Technology, Hangzhou, China; ^2^School of Management, Zhejiang University of Technology, Hangzhou, China

**Keywords:** MNCs, internationalization, neuroeconomics, neuroimaging, cognitive differences

## Abstract

From a classical perspective, multinational companies (MNCs) operate based on market determinants, but recent economic discussions emphasize the central role of the human cognitive system in the decision-making process, thereby giving birth to interdisciplinary fields such as behavioral economics and neuroeconomics. While neuroeconomics is still considered an emerging field, hitherto scant studies have investigated the reflection of cultural experiences on the nervous system. Moreover, the interpretation of cultural diversity in the decision-making process, especially from a neuroeconomic perspective, has not been explored. In response, this study, aperspective research in nature, endeavors to research the neural responses to cultural diversity by contextualizing the neuroeconomic discussion within an MNC framework as one of the best multicultural settings. This study demonstrates that the internationalization process of MNCs is established on the cultural identity and integration of the executive. Through a neuroscientific lens, our findings prove that culture is a fundamental determinant of the cognitive makeup; therefore, cultural diversity in MNCs influences their decision-making process. Moreover, the performance of MNCs is driven by cultural harmonization at the executive level. Finally, the study reveals that the novel field of neuroeconomics is instrumental in identifying cultural intuitions on brain cells and the implications on economic decisions even at a corporate level. The main contribution of this study is the initiation of a multidisciplinary merge of the sociocultural and economic as well as neuroscientific fields, as a research center for MNCs’ decision-making processes.

## Introduction

The contribution of traditional economic models, although still relevant at present (Glimcher, 2003; Camerer and Loewenstein, 2004), has been largely altered by the cognitive investigation of the homos economicus, which has opened a completely new perspective into economic thinking. In the process, researchers have found means to translate behavioral attributes into testable economic variables (Glimcher et al., 2005), thereby expanding the economic concept to an integrative field of interdisciplinary models, providing in-depth and relatively holistic solutions to many scientific questions (Glimcher et al., 2005). However, although the influence of culture on the psychological process is well explored, to the best of our knowledge, the neuroscientific understanding of the cultural diversity influence on the cognitive system, especially the economic decision-making process at the corporate level, remains a gray area (Camerer et al., 2005; Glimcher et al., 2005).

This study aims to fill these gaps by conducting a neuroeconomic investigation to identify the effect of culture on economic decisions in a multicultural environment such as multinational companies (MNCs). Using the cross-cultural context of MNCs, we examined how the advancements of neuroscientific technologies explain the effect of cultural differences on the brain cells and the subsequent implication of the economic decision-making process. Echoing these points, both internal and external cross-cultural immersions of MNCs calls for a profound investigation of the decision-making process, from an individual to a corporate level. Therefore, this study proposes a scientific resolution approach where neuroeconomics is necessary for assessing and resolving cognitive diversities at the leadership level. More specifically, this study addresses the situation by focusing on the cultural diversity within MNCs at the executive level.

The contribution of this study is threefold. First, using the neuroscientific approach, we probed the cultural implications on the cognitive reactions, especially in the decision-making process. Furthermore, these interactions are examined within the context of MNCs as the best cross-cultural setting. Moreover, we investigated the cultural implication in the decision-making process at the executive level, as the decision-making body on the MNCs. Above all, this study is a novel interdisciplinary platform merging culture, economics, and neuroscience, focusing on the initiation of a domain for intriguing discussions involving the three and other similar disciplines.

## Neuroeconomics and Cross-Culture

### Advances in Neuroeconomics

From a historical perspective, the classical market, although run by rational and unemotional subjects, controlled by demand and supply, and driven by profit maximization, has made remarkable contributions to the theoretical and experimental economic landscape (Camerer, 2003, 2005). However, the authors have confirmed the obvious deviations from the traditional economic concept, where, evidentially, economic decision-making was clearly dependent on cognitive and psychological influences. From then, it became a necessity to approach the subject of economic decision-making from a multidisciplinary perspective, forging together economics and psychology to address behavioral economic questions (Camerer et al., 2005). Camerer explained in most of his studies the quest for a better understanding of cognitive processes in decision-making, therefore necessitating a neuroscientific study to further elaborate the rapport between the brain regions and economic behaviors.

Neuroimaging techniques are used to take images of the brain and from color changes of the different regions of the brain to determine corresponding insinuations. Some of the historical brain imaging techniques are electromagnetic recordings and the hemodynamic response method. First, electromagnetic recording techniques include electroencephalography (EEG), which consists of measuring electrical activities in synchrony with stimulus and behavioral reactions using electrodes attached to the scalp (Camerer et al., 2005). A similar and complementary technique is magnetoencephalography (MEG), which is used to determine much more sensitive magnetic field changes in different cortical activities during a decision-making process. Beyond EEG, MEG is used to identify and interpret the deeper activity of the brain structure. Second, the hemodynamic response methods include PET, which measures the blood flow in the brain as an interpretation of neural activities in a specific region of the brain (Camerer et al., 2005; Glimcher et al., 2005), and functional MRI (fMRI), using magnetic property changes due to blood oxygenation to identify the brain morphology with a clear contract in resolution (Glimcher, 2002; Camerer et al., 2005). According to Glimcher (2003), even though these techniques have some strengths and weaknesses, they are used in complementarity to produce more comprehensive results.

In the quest to deepen the understanding of the neural substrates of cognitive functioning, recent studies have adopted a game theory approached using neuroscientific techniques to identify brain cell mechanisms in response to decision-making stimuli. Some strategic formations such as the Ultimatum game, the Consilience game, Prisoner’s Dilemma game, the Cognitive Equilibrium theory, and the Cognitive Hierarchy theory are designed to test and record various aspects of the cognitive makeup and the corresponding reactions (Sanfey et al., 2003; Costa-Gomes and Crawford, 2006). Through the game approach, the neuroimaging technologies reveal that unfair offers provoke reactions in the anterior insula and dorsolateral prefrontal cortex, and the satisfactorily emotional expressions reside in the ventromedial prefrontal cortex of the brain. Significantly, these nervous reactions interpret the specific emotional triggers of the rejection and acceptance of the decision-making processes (Sanfey et al., 2003; Costa-Gomes and Crawford, 2006).

### Cross-Cultural Implication in Economic Decision-Making

The implication of culture on the decision-making process captured by brain imaging technology is best elaborated through the aesthetic triad, which involves the functioning and the interaction of the three large-scale systems (i.e., sensory-motor, knowledge-meaning, and emotion-valuation systems; Vartanian et al., 2015; Chatterjee and Vartanian, 2016). According to fMRI findings, parahippocampal and olfaction of the sensory-motor system are responsible for capturing and remembering, respectively, visual and non-visual aspects of culture (Vartanian et al., 2015), while the grid cells of the hippocampus (knowledge-meaning system) are instrumental for the accumulation of educational and cultural experiences (Chatterjee and Vartanian, 2016). Moreover, PET results have also confirmed the close interaction of two systems with the emotion-valuation systems, working as the decision-making regulator based on the cultural background (Kirk et al., 2009).

Undeniably, the discoveries of brain-imaging technologies, so far, demonstrate the emotional implications of cultural memories on cognitive decision-making, which have a direct influence on preferences (Chatterjee and Vartanian, 2016). In addition to the obvious impact of cultural memories on economic decision-making, the aesthetic triad has also ascertained the phenomenon of the “fear for the unknown,” which explicitly demonstrates the neuroeconomic assessment of the decision-making process (Sanfey et al., 2003). Established on memories, culture is a direct source of emotional and preferential influence of neuroeconomic resolutions, as well as economic decisions determined by the unknown.

### Cross-Cultural and Neuroeconomic Implication in Multinational Companies Decision-Making Process: A Nascent Territory

The literature is explicit on the impact of culture on cognitive reactions and economic decision-making processes; however, the question has not been adequately explored from a neuroeconomic point of view (Shweder, 1991; Camerer et al., 2005). Furthermore, when cultural differences are explained from the perspectives of individual and collective differences, especially cultural diversity being typical and central to MNCs, there is a demand for an elaborate exploration. Again, the understanding of cultural diversity from a neuroscientific perspective is a completely nascent territory, particularly to open a new paradigm for the operation of MNCs for the international market expedition (Shweder, 1991; Chin et al., 2021).

**FIGURE 1:**
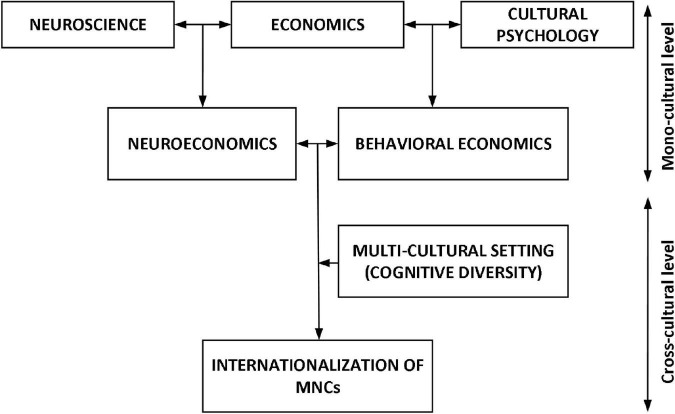
Concept.

With an increasing number of MNCs, neuroeconomic optic could be a standard instrument to interpret the behavior of global investors. Currently, research methodology in neuroscience is shifting from correlational techniques to out-of-sample behavioral predictions (Camerer et al., 2005; Glimcher et al., 2005). Also, much more electric and magnetic brain stimulation methods are being used to manipulate specific regions in order to induce particular behavioral changes. As more light is being shared on the decision-making process in the brain regions, neuroeconomics should be able to tie different cultural interpretations to cognitive intuitions. Motivated by experimenting neuroeconomics for successful MNCs’ internationalization agenda, a cultural diversity dimension is introduced to analyze the cognitive diversity from a neuroeconomic perspective (Figure 1).

In this regard, neuroeconomic discoveries are very instrumental for the economic decision-making process of MNCs, especially in the process of internationalization. Unavoidably, the cognitive diversity of MNCs’ functionaries demands for a neuroeconomic understanding of both the internal and external functionaries. However, the cognitive differences, apart from the national diversity, are importantly the results of cultural diversities.

## Cross-Cultural Cognitive Differences at the Multinational Companies Executive Level: A Neuroeconomic Perspective

Understanding cognitive differences across cultures in MNCs gives assurance for success in a foreign and ambiguous market, as compared to a total dependency on traditional market determinants. With the fast rate of globalization, comes more compounding challenges; therefore, neuroeconomic experimentation is needed for practical implementations (Camerer et al., 2005; Glimcher et al., 2005), especially at the leadership level. Nevertheless, neuroeconomics being an emerging subject calls for more studies, especially the cross-cultural implication at the MNCs’ operational level. In the following sections, we tackled some of the challenging questions of cultural differences facing MNCs and, from a neuroeconomic perspective, explored some effective solutions and the need for further investigations in such areas.

### Knowledge Management in Multinational Companies: The Beginning of the Internationalization Process

As a crucial drive for competitive advantage, knowledge generates value to support the inherent capability of MNCs to improve performance (Chin et al., 2016). Meanwhile, the recent information age and knowledge society have noted an explosion of the knowledge management literature approached from a multidisciplinary perspective, where MNCs can strategically capture, store, use, and share information in order to make knowledge available throughout the company (Chin et al., 2016). Echoing the indispensability of knowledge capturing, storing, using, and sharing for organizational operations, from a cultural psychological perspective, knowledge sharing (KS) is the inborn or acquired behavior of an individual to propagate and transfer knowledge throughout an organization (Chin et al., 2016). Consequently, the internationalization process of MNC begins with management and employee attitude toward KS (Chin et al., 2016). This study proposes a cognitive approach to reveal a cultural mechanism by emphasizing the commitment to KS based on the cultural differences at the executive level. According to the cultural influence theory, the three processes of knowledge conversion, namely, **compliance, identification, and internalization**, characterize the KS process of MNCs’ international decision-making process (Malhotra and Galletta, 2005). According to **compliance**, the motivation mechanism is totally externally sourced. In this case, people’s KS motivation is generally based on rewards or punishments, where they perceive the rewards or punishment consequences to largely outweigh the potential costs (Malhotra and Galletta, 2005). **Identification** is triggered by psychological forces (Bock et al., 2005), where the intention of KS emerges from the desire to maintain and reciprocate interpersonal relationships. Identification is derived from the acknowledgment of others, that is, self-satisfaction from an interpersonal connection with others for knowledge provision and reception (Bock et al., 2005; Chin et al., 2016). While **internalization** is an outcome of the motivation mechanism where people regard KS as a self-worth channel to contribute to the teamwork process and improve the efficiency of the entire community (Chin et al., 2016). Evidently, international motivation starts from an individual cognitive perspective (Malhotra and Galletta, 2005), mostly sourced from cultural identity. Therefore, we argued that the KS attitude of an individual is majorly derived from his community and cultural perspective. Furthermore, in addition to Kelman’s three commitment levels, we deduced an additional level with the new commitment “**conformity**.” This refers to the case where a leader initiates KS by blindly conforming to other leaders’ perspectives at the executive level. For any KS motivation, the four commitment levels can harmoniously synchronize, respectively, according to the situation and different involvements (Chin et al., 2016).

### Multicultural Leadership Context in Multinational Companies

While cultural psychology demonstrates the intrinsic correlation between culture and the brain, neuroscience explains further, by brain mapping, the neural intuitions at different regions of the brain (Shweder, 1991). In the perspective of this “Multicultural Leadership Context in Multinational Companies” section, MNCs will be the primary beneficiaries of further studies on the dynamics of culture and its role in the decision-making body of multicultural MNCs with a common goal. Echoing this, the comparison between cultural groups is conducted alongside, emphasizing people’s differences. It is important not only to examine individual differences but also to investigate the factors that are responsible for the obvious cultural differences and therefore the neural implications in such differences, especially in a cross-cultural context. For this matter, a systematic examination from the neuroeconomic perspective has become critical, so as to further the study on the cognitive variations in terms of cultural individualism and collectivism, especially in MNCs (Chin et al., 2021). For instance, a study comparing European and Asian cultures reveals that the Asian culture emphasizes on “emotional knowledge” while the European culture emphasizes on external behavior rather than inner psychological states (Duan et al., 2021).

To a larger extent, as demonstrated in the literature, the comparison between Western- and non-Western-dominated leadership shows an obvious cultural difference, and how it is managed directly reflects on the organization’s performance (House, 1971, Yukl, 1994). According to the Western cultural concept, an effective leader is one who, through his/her personal competence, can influence subordinates to accomplish goals even beyond their immediate self-interest (House, 1971, Yukl, 1994). Western leadership is task- or relationship-oriented, thus being consistent with the expectation of followers and being able to reach the set target. By the leadership style alone, an individual can be acknowledged by his/her work environment as a leader-making, thereby making it more effective. The Asian leadership concept, in contrast, is entrenched in traditional doctrines. Notably, from the Chinese cultural background, the Confucian Tianxia view is an inward-looking perspective, emphasizing harmony within a team, the Japanese harmony-performance Misumi’s view, and the Indian nurturant task-unity-oriented model of Sinha (Yukl, 1994). From this perspective, leadership is rather sought for inspiration from colleagues and subordinates through a persuasive and coercive approach to promote equality and harmony with others as well as with nature (House, 1971).

Consequently, the leadership cultural diversity could be a major source of distraction and detrimental to the development of MNCs. Therefore, in the midst of the recent highly volatile market, we posited that a scientific approach, such as a neuroeconomic perspective, could be most appropriate to address the multicultural leadership complexity of MNCs. Evidently, neuroeconomic studies adequately disclose, to a deeper extent, the within-group and the within-individual cognitive differentiations, and the end product of individual and cultural interactions proves the diversification of economic decision-making imposed by the cultural diversity within an international context. We argue that a comprehensive application of neuroeconomic measures in the decision-making process of MNCs is very instrumental for bridging the cultural differences, especially at the executive level. The abovementioned game strategies and neuroscience techniques could be introduced at different levels of personnel assessment, in order to ascertain the cultural identity which will be unequivocal for constituting highly efficient team combinations and associations.

### Prospects of Brain Mapping in Multinational Companies Personnel Assessment

Undeniably, the implementation of neuroeconomics in the operation of MNCs is a paradigm shift in the understanding and resolution of cognitive differences, especially within the decision-making leadership characterized by a multicultural formation. Nevertheless, being an emerging discipline, the application of neuroeconomics in addressing the cultural divisions in MNCs necessitates further exploration. Taking the brain to be a “black hole,” every neuroscientific discovery assumes to break off the seal and finally bring in illumination in the entire function of the brain. However, from the intracranial recording and stimulation techniques, to transcranial magnetic stimulation, through electromagnetic recordings, hemodynamic responses to neural activities, and fMRI techniques, it is clear that there are many gray areas yet to uncover. In 2004, Camerer, Loewenstein, and Prelec were the first to write about the neuroeconomic agenda, with the hope that the electromagnetic recording, the hemodynamic response methods, and the new fMRI neuroimaging techniques will be instrumental to identify the different regions of the human brain and their respective involvement in different decisions, therefore revealing the thoughts of people through the activities in the different regions of the brain (Camerer et al., 2005). This hypothesis was reasonable, as neuroscience at that time had already identified neural processes as mentioned above; nevertheless, at present, it is still difficult to identify analogs linking brain regions and psychological identities such as “value, trust, respect, etc….” With time, it becomes even harder to prove this hypothesis because the same brain region is found to be involved with a wide array of tasks. Also, research on brain damage patients (“lesions”) has proven the challenge in identifying connections between parts of the brain and the impairments, despite the assurance on the existence of the connections (Camerer et al., 1989).

From the story of neuroeconomic advancement so far, subsequent discoveries are eminent in near future. It might be possible to establish an estimation evaluation from the weakest strand to the strongest connection between the very neural mechanism and the corresponding behavioral process. For instance, since highly emotional events are remembered vividly, mostly with great details, evidently, it can be hypothesized that connections built on emotions will be robust, more easily identifiable than automatic cognitive intuitions (Camerer et al., 2005; Glimcher et al., 2005).

## Conclusion and Future Direction

Classical economics has laid a firm foundation for the economic field, but subsequent studies demonstrate the impact of cultural and psychological implications in economic decision-making, especially in MNCs where cognitive, cultural diversities are blatantly evident. Consequently, the emancipation or internationalization of MNCs is fundamentally resourced from a cross-disciplinary integration, which gave rise to new research fields such as neuroeconomics. Emerged just about a decade ago, neuroeconomics provides the perfect ground to examine the operation of the brain and the economic decision-making process.

However, the cultural implication in neuroeconomics, as relevant as it is, especially for the operation of MNCs, remains a novel research field. To facilitate a successful internationalization process of MNCs, it is relevant to explore, through a neuroeconomic lens, the intricacies of cognitive difference and cross-cultural influence on the economic decision-making process, especially at the executive level. To well position the discussion, this study highlighted how major discoveries have evolved and given shape to this research field. Discoveries in neuroscience have shed more light on the application of neuroimaging techniques to access brain metabolisms induced from psychological mechanisms and their corresponding behavioral results. In fact, advances in neuroscience are very instrumental to MNCs, especially to reconcile the internationalization decision-making motivation with cognitive diversity across cultures with their corresponding neural intuitions. Therefore, this study proved that the multicultural complexities of MNCs need scientific resolutions, and a neuroeconomic approach is proposed to be instrumental in assessing and resolving cognitive diversities at the leadership level. As an emerging interdisciplinary field, further studies to identify the correlation between the specific regions of the brain with a specific cultural identity and a specific economic decision will be instrumental for the survival of MNCs in economic instabilities.

## Data Availability Statement

The original contributions presented in the study are included in the article/supplementary material, further inquiries can be directed to the corresponding author/s.

## Author Contributions

HC designed and conducted the research and also wrote the first draft of the manuscript. GA searched and analyzed the literature and related data and wrote the main part of the manuscript. TZ provided guidance throughout the entire research process and was also responsible for important review work. HP wrote the remaining parts and offered modification suggestions especially in the aspect of the multicultural context in multinational companies. All authors contributed to the article and approved the submitted version.

## Conflict of Interest

The authors declare that the research was conducted in the absence of any commercial or financial relationships that could be construed as a potential conflict of interest.

## Publisher’s Note

All claims expressed in this article are solely those of the authors and do not necessarily represent those of their affiliated organizations, or those of the publisher, the editors and the reviewers. Any product that may be evaluated in this article, or claim that may be made by its manufacturer, is not guaranteed or endorsed by the publisher.
